# Tae-miR397 Negatively Regulates Wheat Resistance to *Blumeria graminis*

**DOI:** 10.3390/plants12173096

**Published:** 2023-08-29

**Authors:** Yuanyuan Guan, Zhiyuan Wei, Luyi Zhou, Kaige Wang, Meng Zhang, Puwen Song, Ping Hu, Haiyan Hu, Chengwei Li

**Affiliations:** 1 School of Life Sciences, Henan Engineering Research Center of Crop Genome Editing, Henan International Joint Laboratory of Plant Genetic Improvement and Soil Remediation, Henan Institute of Science and Technology, Xinxiang 453003, China; gyy1985@hist.edu.cn (Y.G.); weizy01@126.com (Z.W.); zhouluyi2021@163.com (L.Z.); 17613835385@163.com (K.W.); 2 School of Agriculture, Henan Engineering Research Center of Crop Genome Editing, Henan International Joint Laboratory of Plant Genetic Improvement and Soil Remediation, Henan Institute of Science and Technology, Xinxiang 453003, China; mengz1115@163.com (M.Z.); spw@hist.edu.cn (P.S.); hupingnd@163.com (P.H.); 3 College of Biological Engineering, Henan University of Technology, Zhengzhou 450001, China

**Keywords:** wheat, Tae-miR397, powdery mildew, wound-induced protein

## Abstract

MicroRNA (miRNA) plays a crucial role in the interactions between plants and pathogens, and identifying disease-related miRNAs could help us understand the mechanisms underlying plant disease pathogenesis and breed resistant varieties. However, the role of miRNA in wheat defense responses remains largely unexplored. The miR397 family is highly conserved in plants and involved in plant development and defense response. Therefore, the purpose of this study was to investigate the function of tae-miR397 in wheat resistance to powdery mildew. The expression pattern analysis revealed that tae-miR397 expression was higher in young leaves than in other tissues and was significantly decreased in wheat Bainong207 leaves after *Blumeria graminis* (*Bgt*) infection and chitin treatment. Additionally, the expression of tae-miR397 was significantly down-regulated by salicylic acid and induced under jasmonate treatment. The overexpression of tae-miR397 in common wheat Bainong207 enhanced the wheat’s susceptibility to powdery mildew in the seedling and adult stages. The rate of *Bgt* spore germination and mycelial growth in transgenic wheat plants overexpressing tae-miR397 was faster than in the untransformed wild-type plants. The target gene of tae-miR397 was predicted to be a wound-induced protein (Tae-WIP), and the function was investigated. We demonstrated that silencing of *Tae-WIP* via barley-stripe-mosaic-virus-induced gene silencing enhanced wheat’s susceptibility to powdery mildew. qRT-PCR indicated that tae-miR397 regulated wheat immunity by controlling pathogenesis-related gene expressions. Moreover, the transgenic plants overexpressing tae-miR397 exhibited more tillers than the wild-type plants. This work suggests that tae-miR397 is a negative regulator of resistance against powdery mildew and has great potential for breeding disease-resistant cultivars.

## 1. Introduction

Wheat (*Triticum aestivum* L.) is the main crop grown globally, ensuring food safety [1]. With the world population expanding, there is an ever-growing demand for wheat. Powdery mildew (PM) is one of the most prevalent fungal diseases caused by *Blumeria graminis* f. sp. *tritici* (*Bgt*) [2], which results in a 10–50% reduction in wheat production [3]. The creation of disease-resistant cultivars is an efficient method of controlling wheat disease, and the characterization of resistance genes with excellent agronomic performance is the key to this process. Great efforts have been devoted to identifying genes conferring resistance to powdery mildew [4,5,6], but most of these genes are race-specific, lack resistance or are linked to undesirable traits [7,8,9]. Therefore, new broad-spectrum and robust resistance genes are urgently needed to breed long-lasting disease-resistant wheat cultivars.

MicroRNA (miRNA), a short non-coding RNA with a length of 20–24 nucleotides, plays a significant role in plant development and stress responses [10,11,12]. Many studies have demonstrated that plant miRNAs participate in combatting pathogens by suppressing their targets [13,14,15,16]. For instance, miR482 family members have been shown to adversely influence plants’ resistance to pathogens by targeting specific nucleotide-binding site––leucine-rich repeat (*NBS-LRR*)-family genes in tomato, cotton, and potato [17,18,19,20]. The Csn-miR477/*PAL* (phenylalanine ammonia-lyase gene) module regulates tea plants’ response to disease by restricting the synthesis of secondary metabolisms with an antibacterial effect [21]. In cotton and Chinese white poplar, miR164 plays a critical role in disease defense by degrading NAC transcription factors [22,23]. The miR319/*TCP*, miR394/*LCR* and miR167d/*ARF12* modules have been revealed to be involved in the plant immune response [24,25,26,27]. These studies demonstrate that miRNA is a key player in the interaction between plants and -pathogens via various regulatory mechanisms and a valuable source for improving plant resistance.

The ability of miRNA in wheat stress responses has only been examined in a few studies. It was found that TaemiR408 can affect wheat responses to heavy metal stress, salt stress and stripe rust by targeting a gene encoding a chemocyanin-like protein [28,29]. Another report indicated that miR172 regulated wheat tolerance to salt by targeting INDETERMINATE SPIKELET1 (IDS1) [30]. One hundred thirty-one miRNAs were responsive to wheat yellow mosaic virus via miRNA sequencing [31]. Moreover, numerous miRNAs were discovered to be differently expressed after wheat was inoculated with *B. graminis* and *F. graminearum* [32,33]. Although several potential disease-related miRNAs were discovered in wheat through small RNA sequencing, their roles and regulatory mechanisms are still largely unknown. 

miR397 is a type of ancient and highly conserved miRNA that participates in many important biological processes, including circadian rhythm, flowering, root lignification, fruit development and stress response in plants by silencing the target gene [34,35,36,37,38]. Many recent studies have reported that miR397 contributes to plant responses to pathogens. For example, it was found that miR397 had a negative influence on pathogen resistance by regulating *LAC* gene expression in cotton and malus [39,40]. Our earlier work discovered that sly-miR397 negatively regulated tomato defence against pathogens [41]. These studies suggest that miR397 is an important regulator of plant immune response and could be exploited in resistance breeding. However, no experimental data has been published to date addressing the potential role of tae-miR397 in wheat against pathogens. Therefore, the purpose of this work was to evaluate the role of tae-miR397. 

We found that the expression of tae-miR397 was dramatically decreased after wheat was inoculated with *B. graminis*. Transgenic wheat plants overexpressing tae-miR397 displayed greater susceptibility to *Bgt* than the untransformed WT plants. We also observed that the transgenic plants had more tillers than the WT plants. The BMSV-VIGS method was used to study the function of the target gene of tae-miR397, and the results showed that the silencing of the target decreased powdery mildew resistance in the common wheat Bainong207. In addition to confirming that tae-miR397 negatively regulates wheat’s response to pathogens but positively regulates tiller development, our study also provides a valuable gene source for improving disease resistance in common wheat. It sheds light on the mechanism of tae-miR397 in regulating wheat disease resistance.

## 2. Results

### 2.1. The Phylogenetic Relationship of miR397 and the Structure of tae-miR397

**Figure 1:**
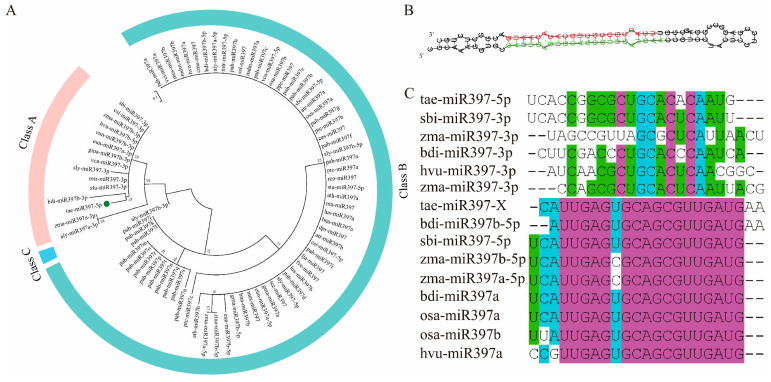
Phylogenetic relationship analysis of miR397 and second structure of tae-miR397 precursor. (**A**) The phylogenetic tree was built using the Maximum likelihood method with 1000 bootstrap replicates in MEGA 7.0. (**B**) Prediction of the secondary structure of the precursor tae-miR397. Base-in red indicates the mature sequence of miR397-5p; Base-in green indicates the mature sequence of miR397-X. (**C**) Alignment of mature miR397 in grass species.

There were 84 mature members and 71 precursors of miR397 family members in 33 species documented in miRBase. To study the evolutionary relationships of the miR397 family members, mature sequences were used to build a phylogenetic tree with MEGA 7.0 and classified into three classes (class A, B, C and D) (Figure 1A), while the miR397 precursors were divided into four classes (Figure S1), indicating that the miR397 precursors were less conservative than mature miR397 members. Most mature miR397-3p members were clustered into class A, and the other mature miR397 family members were clustered into class B. Aly-miR397-3p were classed into C. The number of mature miR397 members in class B was larger than in class A, indicating that most mature miR397 members in different plant species are formed from the 5′ arm of miR397 precursors. The mature miR397-3p members in class A were quite similar to each other and clustered into two subclasses, while mature miR397 members in class B were less conservative and clustered into five subclasses. 

The secondary structure pre-tae-miR397 was predicted, and it can form a stable stem-loop structure (Figure 1B). The mature sequence of tae-miR397 is located at the 3′ arm of the pre-tae-miRNA397 (shown in red in Figure 1B), which is, however, identified as miR397-5p in miRBase. The matching sequence at the 5′ arm of the tae-miR397 precursor was CAUUGAGUGCAGCGUUGAUGAA (shown in green in Figure 1B), which was named tae-miR397-X in the present study but not recorded in miRBase. The sequences of the mature miR397 in *Gramineae* were aligned, and it was found that the sequences of miR397-5p were highly conserved, whereas the sequences of miR397-3p were relatively divergent. The sequence of tae-miR397-X is nearly identical to that of miR397-5p from the other plants, whereas the mature tae-miR397-5p is identical to miR397-3p in other grass species (Figure 1C), suggesting that miR397-5p may have been named incorrectly in the previous record.

### 2.2. Expression Patterns of tae-miR397 

**Figure 2:**
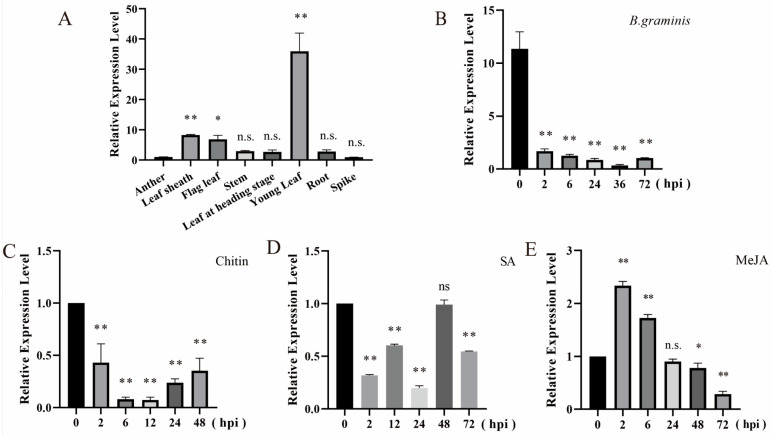
Expression pattern of tae-miR397. (**A**) Expression pattern of tae-miR397 in wheat issues. (**B**) Expression pattern of tae-miR397 in wheat variety Bainong207 seedling leaves inoculated with *Bgt*. (**C**) Expression pattern of tae-miR397 in wheat variety Bainong207 seedling leaves after chitin treatment. (**D**) Expression pattern of tae-miR397 in wheat variety Bainong207 seedling leaves after SA treatment. SA, salicylic acid. (**E**) Expression pattern of tae-miR397 in wheat variety Bainong207 seedling leaves after MeJA treatment. MeJA, methyl jasmonate. Relative expression levels are representative of the mean values of three biological replicates. Error bars represent one standard deviation (SD). The * and ** represent significant differences at the levels of *p* < 0.05 and *p* < 0.01 between the control and treatment groups using Tukey’s multiple comparisons test.

The expression patterns of tae-miR397 in different tissues were examined via qRT-PCR. The results revealed that tae-miR397 was ubiquitously expressed in leaves, roots, stems, leaf sheathes, anthers and spikes, with the highest expression in young leaves (Figure 2A).

To evaluate whether tae-miR397 could respond to pathogen infection, wheat leaves were inoculated with *Bgt* fungus and subjected to plant hormone and chitin treatments, and qRT-PCR was performed to examine the expression level of tae-miR397. The results showed that the accumulation of tae-miR397 was strongly down-regulated and reached its lowest level at 36 hpi after *B. graminis* inoculation (Figure 2B). Similarly, the transcription levels of tae-miR397 were significantly decreased after chitin treatment (Figure 2C). SA and MeJA were applied to the seedlings of wheat variety Bainong207. After SA treatment, the accumulation of mature tae-miR397 was significantly down-regulated and lowest at 24 hpi (Figure 2D). In contrast, the expression level of tae-miR397 was increased after MeJA treatment, but the fold change was small (Figure 2E). These results showed that tae-miR397 could respond to *Bgt* fungus and resistance-related hormone treatment and might play a role in disease resistance via SA-mediated signaling pathways. 

### 2.3. Overexpression of tae-miR397 Reduces Wheat Resistance against B. graminis 

**Figure 3:**
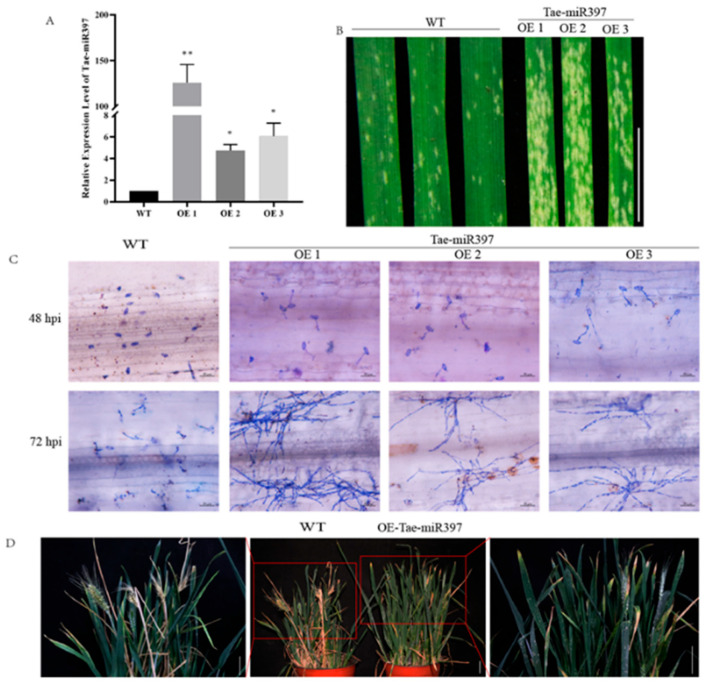
Overexpression of tae-miR397 reduces wheat resistance against *B. graminis* (**A**) Expression levels of tae-miR397 in miR397-OE plants and WT plants. The * and ** represent significant differences between WT and transgenic plants at *p* < 0.05 and *p* < 0.01 using Tukey’s multiple comparisons test. (**B**) The disease phenotype of detached leaves from miR397-OE plants and the control Bainong207 inoculated with *Bgt* at the seedling stage. Bar: 3 cm (**C**) Microscopic observation of *Bgt* development in miR397-OE plants and WT plants leaves inoculated with *Bgt* fungus for 48 and 72 h. Bar: 50 μm. (**D**) The disease phenotype of whole adult plants inoculated with *Bgt* in the heading stage. Bar: 5 cm; WT: Wild-type wheat variety Bainong207; OE: Transgenic plants overexpressing tae-miR397.

To elucidate the function of tae-miR397 in wheat against *B. graminis*, wheat plants overexpressing miR397 (miR397-OE) were generated. Positive transgenic plants were identified using GUS staining, and leaves dyed blue were defined as putative positive lines (Figure S2), which were then confirmed via qRT-PCR. The relative expression levels of tae-miR397 in transgenic wheat plants were considerably higher than those in WT plants and increased by approximately 125, 3.7, and 5 times (Figure 3A), showing that overexpression of tae-miR397 did occur. For the powdery mildew resistance evaluation, the detached leaves taken from these three transgenic lines in the seedling stage were cut and subsequently inoculated with *Bgt* spores. The disease symptoms in the miR397-OE plants were more severe than those in WT plants at 7 dpi (Figure 3B). The developmental phenotypes of spore germination and mycelial growth were observed under the microscope. The spore germination rate in the transgenic plants was faster than that in the WT plants at 48 hpi. At 72 hpi, the hypha mass was much greater in the overexpression plants than in WT plants (Figure 3C), which was in line with disease symptoms in detached leaves. miR397-OE plants were also inoculated with *Bgt* spores in the adult stage and displayed greater vulnerability to powdery mildew than WT plants (Figure 3D). These results indicated that tae-miR397 adversely affected wheat resistance to *B. graminis* during the entire growth stage.

### 2.4. Overexpression of tae-miR397 Decreases Pathogenesis-Related Gene (PR) Expression

**Figure 4:**
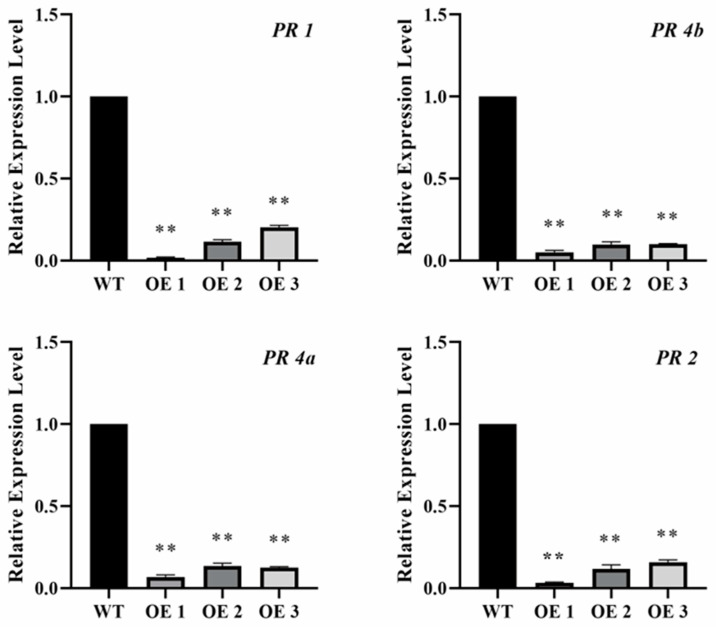
Relative expression levels of *PR* genes in seedlings with *Bgt* inoculation. The relative expression levels of four *PR* genes are representative of the mean values of three biological replicates. The ** represent significant differences between WT and transgenic plants inoculated with *Bgt* at the levels of *p* < 0.05 and *p* < 0.01 using Tukey’s multiple comparisons test. WT: Wild type wheat variety Bainong207. OE: Transgenic plants overexpressing tae-miR397.

The *PR* gene is crucial for the plant’s defense against fungal pathogens. To ascertain whether tae-miR397 regulates the wheat-pathogen interaction by influencing *PR* gene expressions, *TaPR1*, *TaPR2*, *TaPR4A* and *TaPR4B* were chosen to analyze their expression levels in miR397-OE and WT plant leaves infected with *Bgt*. The transcription levels of all four selected *PR* genes were considerably lower in miR397-OE seedlings than in WT plants (Figure 4), implying that tae-miR397 negatively regulated wheat resistance to powdery mildew by downregulating the expression of *PR* genes.

### 2.5. Overexpression of tae-miR397 Increases the Number of Wheat Tillers 

**Figure 5:**
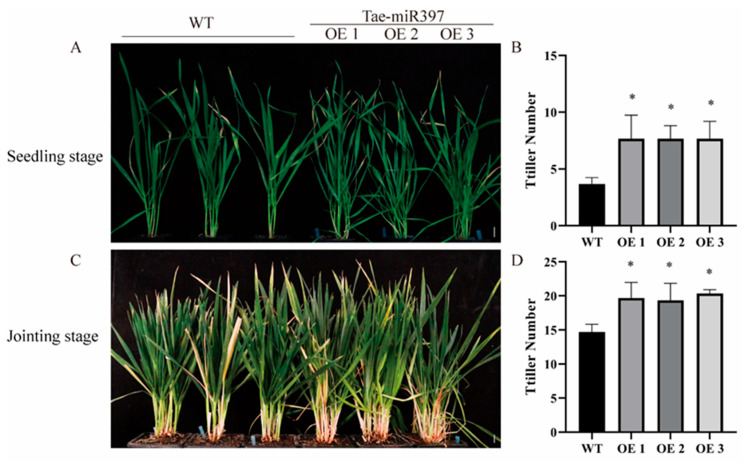
Tillers development of transgenic wheat plants overexpressing tae-miR397. (**A**) Tiller phenotypes of miR397-OE plants and WT plants in the tillering stage. (**B**) The number of tillers in the seedling stage. (**C**) Tiller phenotypes of miR397-OE plants and WT plants at the jointing stage. Bar: 2 cm. (**D**) The number of tillers in the jointing stage. The * represent significant differences between WT and transgenic plants at the levels of *p* < 0.05 and *p* < 0.01 using Tukey’s multiple comparisons test. WT: Wild-type wheat variety Bainong207. OE: Transgenic plants overexpressing tae-miR397.

It is well known that miR397 plays an important role in regulating plant growth and development. Transgenic lines overexpressing tae-miR397 in T_2_ and T_3_ generations were used for phenotypic observation in different developmental stages. We found only differences in the number of tillers between transgenic and WT plants during the development process. In the tillering stage, miR397-OE plants showed a considerable increase in tillers compared to wild-type Bainong207 plants (Figure 5A), with an increase of approximately 2.3 tillers (Figure 5B). In the jointing stage, miR397-OE transgenic lines still developed more tillers than the wild-type wheat Bainong207 (Figure 5C), with an increase of approximately three tillers (Figure 5D). These results indicated that tae-miR397 plays a positive role in promoting wheat tillering.

### 2.6. The silencing of Target Genes Reduces Wheat Resistance to Powdery Mildew 

To gain greater insight into the regulatory mechanism of tae-miR397, the target gene was predicted online using tae-miR397-5p and tae-miR397-X as queries. *TraesCS6A02G134500* was selected as the candidate target gene (Expected value = 0) (Table S2), which encodes a hypothetical protein with an unidentified function. We found that TraesCS6A02G134500 contained a conserved DUF3774 domain by searching the Conserved Domain Database (CDD) in NCBI (https://www.ncbi.nlm.nih.gov/Structure/cdd/wrpsb.cgi, accessed on 18 March 2022) (Figure S3), which was present in the majority of wound-induced proteins. Thus, we named TraesCS6A02G134500 as Tae-WIP. The expression pattern of *Tae-WIP* was determined in wheat Bainong207 leaves inoculated with *Bgt*, and it was found that the expression of *Tae-WIP* was dramatically increased after *Bgt* infection (Figure S4), which was contrary to the expressing trend of tae-miR397. Therefore, we concluded that *Tae-WIP* is a potential target gene of tae-miR397.

**Figure 6:**
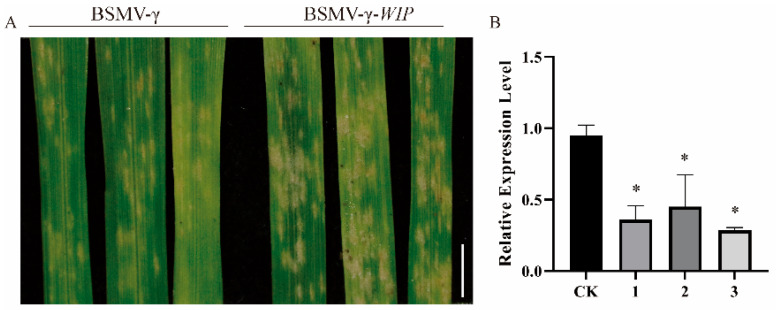
Functional analysis of Tae-WIP using BSMV-VIGS in Bainong207. (**A**) BSMV: WIP-infected plants were inoculated with *Bgt*, and their leaves were photographed at 7 dpi. BSMV:γ infected plants were used as controls. Bar: 1 cm. The experiment was repeated independently three times, and the same results were obtained. (**B**) Expression levels of *Tae-WIP* in BSMV:WIP-infected and BSMV:γ-infected leaves. CK represents plants inoculated with BSMV:γ, and 1–3 represents wheat Bainong207 plants inoculated with BSMV:WIP. The * represents significant differences between plants inoculated with BSMV:γ and plants inoculated with BSMV:WIP at the level of *p* < 0.05 using Tukey’s multiple comparisons test.

The target gene *Tae-WIP* was silenced using a BSMV-based VIGS approach to investigate its function. The leaves inoculated with both the BSMV:γ virus, BSMV:WIP and the BSMV:PDS viruses exhibited chlorotic stripes, and no symptoms were observed on the leaves without virus inoculation. To explore the role of *Tae-WIP* in conferring resistance to *Bgt*, the fourth leaf with obvious virus virulence was inoculated with *Bgt*, and the disease phenotype was photographed at 7 dpi. It was observed that the *Tae-WIP*-silenced groups had more spores than the control groups (Figure 6A). The expression levels of *Tae-WIP* were assessed via qRT-PCR and found to significantly decrease by approximately 3-fold in three different WIP-silenced leaves (Figure 6B), indicating high silencing efficiency. The results outlined above showed that the enhanced susceptibility to *Bgt* was correlated with reduced *Tae-WIP* expressions, which is consistent with the loss-of-resistance phenotype of plants overexpressing tae-miR397 in wheat plants.

## 3. Discussion

Accumulated evidence has highlighted the importance of miRNA in plant-pathogen interactions [42,43]. The role of miRNAs in wheat-pathogen interactions remains largely unknown, and the characterization of resistance-related miRNAs will be helpful for analyzing the regulatory mechanisms of wheat-pathogen interactions and breeding disease-resistant wheat varieties. The expression level of miRNA changed when pathogens infect wheat plants [31,44]) and altering the expression of miRNA could influence plant immunity [45,46], indicating that there is a strong relationship between the degree of miRNA and plant resistance. In the present study, tae-miR397 expression in wheat leaves was found to be significantly decreased after *Bgt* infection. Plants can sense chitin to initiate an innate defense against fungus infections [47]. In particular, the expression of tae-miR397 was rapidly decreased upon chitin treatment. It is generally known that JA and SA play essential roles in defending plants against biotic stressors [15,48,49]. We found that the expression level of tae-miR397 was downregulated by SA but upregulated by JA, consistent with a previous study showing that SA and JA play critical roles in plant defense against biotrophic and necrotrophic pathogens. The SA-mediated pathway is antagonistic to the JA-mediated pathway [50]. The above findings suggested that tae-miR397 may play an important role in wheat response to pathogen infections. Moreover, miR397 members have been shown to be involved in plant immune responses in several plants [39,40,51]. Hence, in this study, we mainly study the function of tae-miR397 in regulating wheat disease resistance via bioinformatics analysis and biotechnology methods.

Bioinformatics analysis showed that the number of miR397 family members varies among plant species. For instance, there are twenty-one members in *Picea abies*, two in *Oryza sativa* and *Zea mays*, and only one in wheat (miRBase: https://www.mirbase.org/, 18 March 2022). In the current work, the phylogenetic analysis showed that the mature sequence of miR397 is highly conserved across different plant species. It is known that a pair of mature miRNAs (miRNA-3p and miRNA-5p) are typically produced when processing a hairpin precursor. Mature sequences of miR397-3p in different plants displayed more isoforms than the mature miR397-5p, which is consistent with previous findings showing that mature miRNA generated from the 3′ arm of the precursor is more variable than that of the 5′ arm [52,53]. Therefore, we concluded that mature miR397-3p may be the driving force during the evolution.

Additionally, the diversity of miR397-3p results in the targeting of diverse genes in accordance with their multiple functions in plant biological processes [54]. Unexpectedly, the mature tae-miR397-5p recorded in the miRBase is located in the 3′ arm of the tae-miR397 precursors, which is, however, quite similar to the miR397-3p sequence in other plant species. Moreover, the corresponding sequence located in the 5′ arm of tae-miR397 shares a great deal of similarities with miR397-5p in other plant species. Here, we discovered that only tae-miR397-5p from the 3′ arm of the precursor was recorded in miRbase, while the corresponding mature sequence tae-miR397-X located at the 5′ arm is not recorded in miRbase, and it has not been detected by high-throughput sequencing [33]. These findings are consistent with previous studies reporting that one end of the mature miRNA product is expressed and functional. In contrast, the other is an unfunctional by-product of the biogenesis process [55]. In conclusion, we questioned whether the tae-miR397-5p (recorded in miRBase) should be regarded as tae-miR397-3p that is functional, whereas the tae-miR397-X (named by us) should be regarded as tae-miR397-5p that is not expressed or non-functional. 

In this study, transgenic plants overexpressing tae-miR397 were constructed to analyze the function tae-miR397, and transgenic lines overexpressing tae-miR397 were generated. In the detached-leaf *Bgt* inoculation assay, the transgenic lines displayed more severe disease symptoms than the WT plants, and spore germination was enhanced in the transgenic plants. This susceptibility may be because transgenic plants promote the penetration of *Bgt* fungus, thereby affecting the infection rate. Subsequently, we observed that the whole transgenic plants exhibited high susceptibility to powdery mildew in the adult stage. These findings indicated that tae-miR397 is a susceptibility gene that has a negative effect on wheat disease resistance throughout the entire growing period, which is consistent with the results of previous studies [39,40,51]. Many studies have demonstrated that the disruption of susceptibility genes is critical for the development of varieties with long-lasting disease resistance [56,57]. Therefore, we theorized that knocking out of tae-miR397 could be performed to obtain an ideal powdery-mildew-resistant variety to prevent *Bgt* infection. 

MiRNAs regulate plant development and stress response by silencing their target genes. To ascertain the regulatory mechanism of tae-miR397 in wheat-pathogen interactions, the analysis of the function of the target gene of tae-miR397 is essential. After bioinformatic analysis, *TraseCS6A02g134500* was predicted to be the target, which is consistent with the results of other investigations [58]. Bioinformatic analysis showed that *TraseCS6A02g134500* encoded an unknown protein with a conserved domain typically present in wound-induced proteins (WIP). It has been reported that WIP positively regulates the *Arabidopsis* plant’s resistance to pathogens [59]. In this study, the BMSV-VIGS method was used to examine the function of the target, and the silencing of Tae-WIP did produce a consistent resistance phenotype with that of overexpression tae-miR397. In addition, WIPs were reported to be able to activate pathogen-associated molecular pattern (PAMP)-triggered immunity (PTI) [59]. Hence, we analyzed the expression levels of *PR* genes to better understand the regulatory mechanism of Tae-WIP through PTI. The qRT-PCR analysis showed that *TaPR1*, *TaPR2* and *TaPR4* were lower in miR397-OE transgenic plants than in WT plants after inoculation with *Bgt*. These results suggest that tae-miR397 contributes to basal resistance against powdery mildew, most likely by knocking down *Tae-WIP* expressions and controlling *PR* gene expressions. In the previous literatures, laccases genes, CKB3, and other unconfirmed genes (*RRA1*/*2*, *DPA*, *CLPP3* and *GNS1*/*SUR4*) have usually been identified as miR397 targets [34,39,60], while in the present study, we found that a wound-induced protein might be the target of tae-miR397.

Several studies report that miR397 participates in plant growth and development [51]. In this study, we noted that the overexpression of tae-miR397 led to the production of more tillers than wild-type wheat plants under greenhouse circumstances. This finding could be used to improve agronomic traits. According to previous studies, overexpression of miR397 promotes rice panicle development and banana biomass by regulating the brassinosteroid-dependent signaling pathway [61]. Does the tae-miR397 regulating tiller number depend on the hormone signaling pathway? We will investigate this question in a future study.

In conclusion, our study showed that tae-miR397 is a susceptibility miRNA and negatively regulates wheat resistance, most likely by silencing *Tae-WIP* and regulating *PR* gene expression. The tae-miR397/WIP regulatory pathway revealed in this study expands our understanding of the regulatory mechanism of miR397. From a practical standpoint, knocking out tae-miR397 by genome editing will endow plants with long-lasting disease resistance and provide a new method to breed high and durable resistance varieties. 

## 4. Materials and Methods

### 4.1. Plant and Fungal Materials Culture

The common wheat (*Triticum aestivum* L.) cultivar Bainong207 (with moderate resistance to powdery mildew) was employed for the pre-miRNA cloning, transformation, and functional analysis of tae-miR397 and maintained at the Henan Institute of Science and Technology (Xinxiang, China). The cultivar Sumai3 (high susceptibility to powdery mildew) [62] was used to maintain *Bgt*. All plants were grown in the controlled chambers at 22 °C with a 16-h/8-h light/dark photoperiod. 

The mixed races of *B. graminis* used in this study were isolated in a wheat field (Henan, China) and maintained on Sumai3 seedlings in an incubator under 22 °C/16-h-light and 18 °C/8-h-dark conditions with approximately 70% relative humidity. 

### 4.2. Analysis of the Expression Pattern of tae-miR397

Leaves, roots, leaf sheathes, stems, anthers and spikes were collected to determine the tae-miR397 expression in different tissues. The expression patterns of tae-miR397 were analyzed in the leaves upon *B. graminis* infection. The leaves of Bainong207 were inoculated with *Bgt* when the seedlings reached the three-leaf stage, which was harvested at 2, 6, 24, 36 and 72 h post-inoculation (hpi). 100 μg/L chitin, 100 mM methyl jasmonate (MeJA) and 5 mM salicylic acid (SA) were sprayed onto the wheat Bainong207 seedling leaves. The water-sprayed plants were served as controls. The leaves were harvested at different times after each treatment. All samples were immediately frozen in liquid nitrogen and kept at −80 °C for total RNA extraction and qRT-PCR analysis. Three independent biological replicates were performed for each treatment. Each treatment was repeated three times.

### 4.3. Production of Transgenic Plants Overexpressing tae-miR397

The 368 bp fragment containing the sequence of the precursor miR397 (pre-miR397) (miRBase accession: MI0030405) and its flanking sequences were amplified from wheat genomic DNA via PCR and then cloned into the vector pMD18-T for sequencing. The PCR products were subcloned into the vector pCambia1301 using *Bam*H I and *Kpn* I digestion to produce the recombinant plasmid pCambia1301:miR397, which was transformed into wheat Bainong207 using *Agrobacterium*-mediated genetic transformation as described by Wang et al., 2014 [63]. Since the vector pCambia1301 contained the glucuronidase (GUS) reporter gene, candidate-positive transgenic lines were examined using GUS staining. The GUS staining solution was purchased from a company (Hua Yue-yang, Beijing). The leaves were cut and immersed in the GUS staining solution overnight at 37 °C in the dark before being decolorized with 75% alcohol. Positive transgenic plants in the T_1_, T_2_ and T_3_ generations were selected and confirmed by quantifying the expression levels tae-miR397 using qRT-PCR. All primers are listed in Supplementary Table S1.

### 4.4. Resistance Analysis

*Bgt* resistance was analyzed by infecting detached leaves at the seedling stage and whole plants in the adult stage. The fourth expanded detached leaves of miR397-OE plants and WT plants were cut into two sections placed on agar medium containing 20 mg/L 6-BA (benzimidazole) and then inoculated with mixed *Bgt* races under the conditions of 22 °C/16 h of light and 18 °C/8 h of darkness for seven days [64]. One of the two infected sections was collected to determine the expression of pathogenesis-related genes (*PR*) using qRT-PCR. An evaluation of *Bgt* resistance in adult plants was performed in the heading stage, and the phenotype of the disease symptom was photographed at 7 dpi. 

To visualize the spore germination and mycelial development, *Bgt-infected* leaves were collected at 48 and 72 hpi and stained with Coomassie Brilliant Blue, as described by Sánchez-Martín et al. (2021) [65]. Bright-field images were captured using a microscope.

### 4.5. BMSV-VIGS

The target gene of tae-miR397 was predicted to be TraseCS6A02G134500 using psRNATarget (http://plantgrn.noble.org/psRNATarget/, 18 March 2022) and named *Tae-WIP*. *TaeWIP* has three copies scattered in 6A, 6B and 6D genomes, including *TraseCS6A02G134500*, *TraseCS6B02G162700* and *TraseCS6D02G123700*. Because of the high sequence similarity of the three copy genes in different genomes, we cloned the common sequence of the three genes for silencing. The BSMV-VIGS system was used to knock down the transcript level of the target gene. The BSMV-mediated gene silencing in wheat leaves was carried out as described by Yuan et al., 2011 [66]. The 277 bp fragment of the target gene (from +66 to +342) was amplified using primers and cloned into the BSMV:γ vector via homogenous recombination to generate the recombination vector BSMV:WIP. BSMV:TaPDS and BSMV:γ vector were employed as controls. The fully expanded second leaves of the wheat Bainong207 were infected with vitro-transcribed (mMESSAGEmMACHINE T7, Invitrogen, Waltham, MA, USA) viruses BSMV:WIP, BSMV:TaPDS and BSMV:γ. The completely expanded fourth leaves with apparent virus feature after 15 days of infection were collected for inoculation with *Bgt,* and the measurement of silencing efficiency was performed using qRT-PCR. The phenotype of disease symptoms was recorded at 7 dpi. All primers are listed in Supplementary Table S1.

### 4.6. Quantitative Reverse Transcription PCR (qRT-PCR)

Total RNA was extracted from wheat leaves using TRIzol regent (Invitrogen, USA). cDNA (miRNA and mRNA) was synthesized using Mir-X™ miRNA First-Strand Synthesis Kit (Clontech, Fitchburg, WI, USA) and PrimeScript RT Reagent Kit with gDNA Eraser (Takara Bio Inc., Shiga, Japan) respectively, according to manufacturers’ instructions, to quantify the expression levels of miRNAs and mRNAs. The qRT-PCR experiment was performed using TB Green^®^ Premix Ex Taq™ II (Tli RNaseH Plus) (Takara Bio Inc) on the Applied Biosystems 2720 platform (Thermo Fisher Scientific, Waltham, MA, USA). The *tubulin* gene was used as an internal reference control. The 2^−ΔΔCT^ method was used to calculate the relative expression levels of miRNAs and genes [67]. Three independent replicates of each experiment were statistically analyzed. Each experiment was repeated three times. Specific primers for miRNAs, genes and *tubulin* are listed in Supplementary Table S1.

### 4.7. Phylogenetic Analyses and Second Structure of miR397 Prediction

All the mature miR397 and miR397 precursors were downloaded in miRBase (https://www.mirbase.org/) and aligned by ClustalX with the default options in MEGA 7, and a phylogenetictree was constructed using the Maximum Likelihood method with 1000 bootstrap replicates. The secondary structure pre-tae-miR397 was predicted on the RNAfold WebServer (http://rna.tbi.univie.ac.at/cgi bin/RNAWebSuite/RNAfold.cgi, 18 March 2022).

### 4.8. Statistical Analysis

Microsoft Excel was used to determine sample averages and standard errors. Tukey’s multiple comparison tests were used to examine significant differences between different samples.

## Data Availability

Not applicable.

## Supplementary Materials

The following supporting information can be downloaded at: https://www.mdpi.com/article/10.3390/plants12173096/s1, Figure S1: Phylogenetic relationship analysis of pre-miR397. The phylogenetic tree was built using the Maximum likelihood method with 1000 bootstrapreplicates by MEGA 7.0; Figure S2: Positive transgenic plants selected by GUS staining. (A) GUS staining of the transgenic plants overexpressing tae-miR397. (B–C) Leaves dyedblue were candidate positive transgenic plants. (D) WT leaves dyed in GUS staining; Figure S3: Prediction of conserved domain of target gene (WIP) online; Figure S4: Expression pattern of target gene (Tae-WIP) in wheat plants after inoculation of *Bgt.* Relative expression levels are representative of the mean values of three biological replicates. Error bars represent one standard deviation (SD). The * and ** represent significant differences at levels of *p* < 0.05 and *p* < 0.01 between the control and treatment groups using Tukey’s multiple comparisons test; Table S1. Primers used in the study; Table S2. Target genes prediction.
