# Supplementary material for: Tae-miR397 Negatively Regulates Wheat Resistance to Blumeria graminis
Source: Plants (Basel). 2023 Aug 29;12(17):3096. doi: 10.3390/plants12173096 (PMC10489981; doi:10.3390/plants12173096)
Supplement: Supplementary file 1 [file plants-12-03096-s001.zip › plants-2553364-supplementary.pdf]

plants

MDPI

Table S1. Primers used in the study.

Primers	Sequence (5'-3')	objective
qTae-miR397	TCACCGGCGCTGCACACAATG	qRT-PCR
qTatubulin-F	ATCTCCAACTCCACCAGTGTCG	qRT-PCR
qTatubulin-R	TCATCGCCCTCATCACCGTC	qRT-PCR
qTae-WIP-F	GCGCAGACGCAGAGAGAAAG	qRT-PCR
qTae-WIP-R	GTCCTTGAGCGCCTCCAC	qRT-PCR
TaPR1-F	GAGAATGCAGACGCCCAAGC	qRT-PCR
TaPR1-R	CTGGAGCTTGCAGTCGTTGATC	qRT-PCR
TaPR2-F	AGGATGTTGCTTCCATGTTTGCCG	qRT-PCR
TaPR2-R	AAGTAGATGCGCATGCCGTTGATG	qRT-PCR
TaPR4A-F	CGTCTTCACCAAGATCGACA	qRT-PCR
TaPR4A-R	GGCAGTCGACGAACTGGTA	qRT-PCR
TaPR4B-F	CTTCACCAAGATCGACACCA	qRT-PCR
TaPR4B-R	AGCAAGCTAGCCTTTGATCG	qRT-PCR
VIGS-WIP-F	<u>TAGCTGAGCGGCCGCCCCGGG</u>GTGCGCCTTG CGCAGCTTCG	Make silence of WIP
VIGS-WIP-R	<u>TAGCTGATTAATTAACCCGGG</u>GCAAGGGGAG-GAACAGGATC	Make silence of WIP
OTaemiR397-F	<u>GGATCC</u>ACACCTCATCATACTACTAC	Make overexpression of miR397
OTaemiR397-R	<u>GGTACC</u>AACTGAGCTCCTTCTCTCCG	Make overexpression of miR397

Note: The underlined bases are restriction enzyme sites or adaptor sequence.

Table S2. Target genes prediction.

miRNA ACC.	Target Acc.	Expect	UPE	Alignment
Using tae-miR397-5p as request				
UCACCGGCGCUGCACACAAUG	TraesCS6A02G134500.1	0.0	N/A	miRNA 21 GUAACACACGUCGCGGCCACU 1<br>Target 504 CAUUGUGUGCAGCGCCGGUGA 524
UCACCGGCGCUGCACACAAUG	TraesCS7D02G230400.1	1.5	N/A	miRNA 21 GUAACACACGUCGCGGCCACU 1<br>Target 287 GCUUGUGCGCGGCGCCGGUGA 307
UCACCGGCGCUGCACACAAUG	TraesCS2B02G406200.1	2.5	N/A	miRNA 21 GUAACACACGUCGCGGCCACU 1<br>Target 1593 GGUUGUGCGCAGUGCCGGUGC 1613
UCACCGGCGCUGCACACAAUG	TraesCS2A02G299200.1	2.5	N/A	miRNA 21 GUAACACACGUCGCGGCCACU 1<br>Target 179 ACUUGUUUGCGGUGUCGGUGA 199
Using tae-miR397-X as request				
CAUUGAGUGCAGCGUUGAUGAA	TraesCS6A02G134500.1	0.0	N/A	miRNA 22 AAGUAGUUGCGACGUGAGUUAC 1<br>Target 554 UUCAUCAACGCUGCACUCAAUG 575
CAUUGAGUGCAGCGUUGAUGAA	TraesCS1D02G283000.1	2.0	N/A	miRNA 22 AAGUAGUUGCGACGUGAGUUAC 1<br>Target 744 CUCAUCAACGCUGCGCUCAACG 765
CAUUGAGUGCAGCGUUGAUGAA	TraesCS4A02G096400.1	2.0	N/A	miRNA 22 AAGUAGUUGCGACGUGAGUUAC 1<br>Target 772 CUGAUCAACGCUGCGCUCAACG 793
CAUUGAGUGCAGCGUUGAUGAA	TraesCS4D02G208900.1	2.0	N/A	miRNA 22 AAGUAGUUGCGACGUGAGUUAC 1<br>Target 767 CUGAUCAACGCUGCGCUCAACG 788

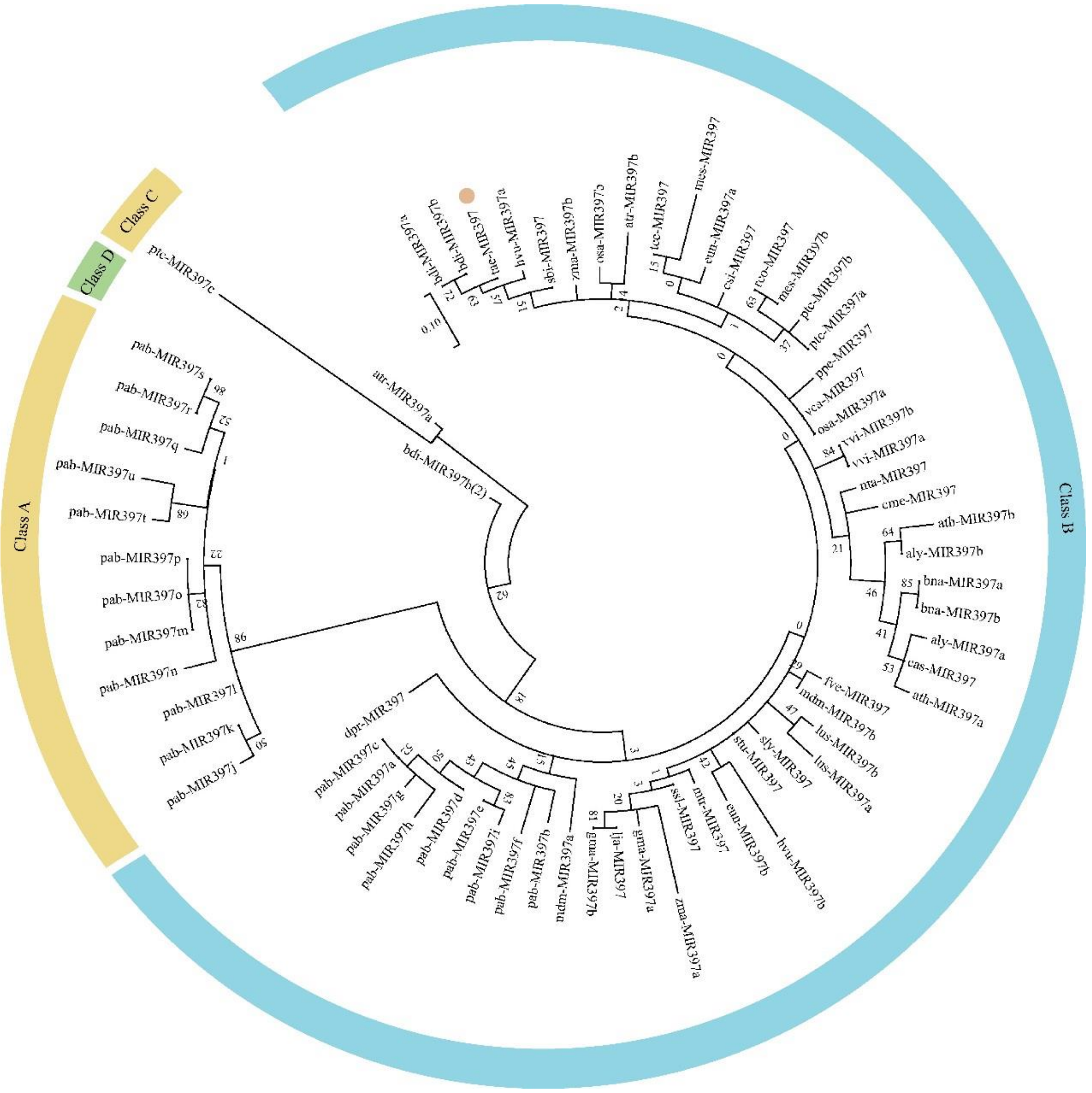

Figure S1. Phylogenetic relationship analysis of pre-miR397. The phylogenetic tree was built using the Maximum likelihood method with 1,000 bootstrapreplicates by MEGA 7.0.

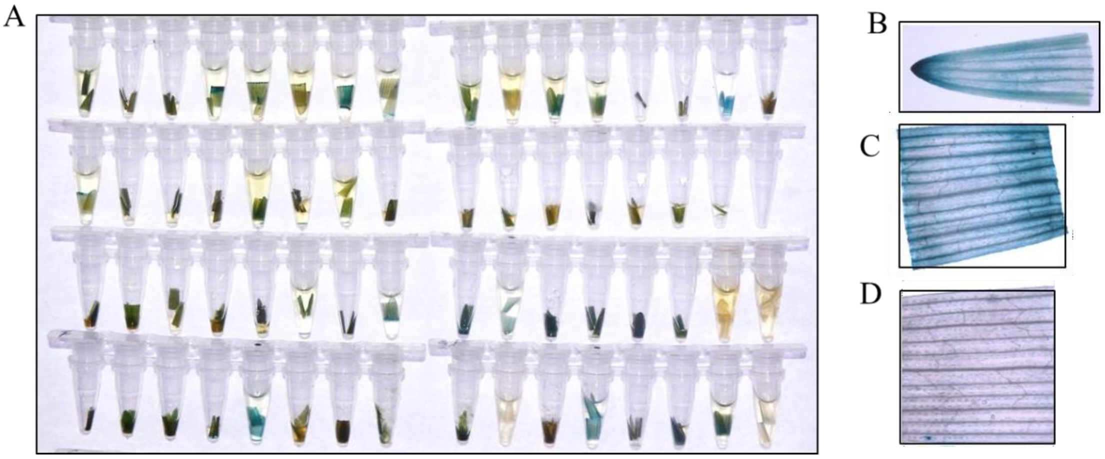

Figure S2. Positive transgenic plants selected by GUS staining. (**A**) GUS staining of the transgenic plants overexpressing tae-miR397. (**B**–**C**) Leaves dyedblue were candidate positive transgenic plants. (**D**) WT leaves dyed in GUS staining.

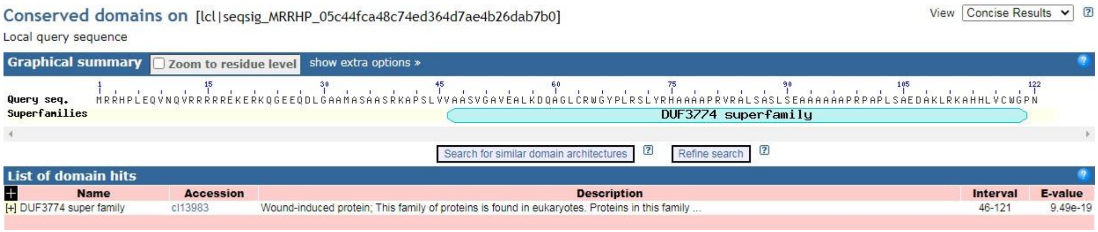

Figure S3. Prediction of conserved domain of target gene (WIP) online.

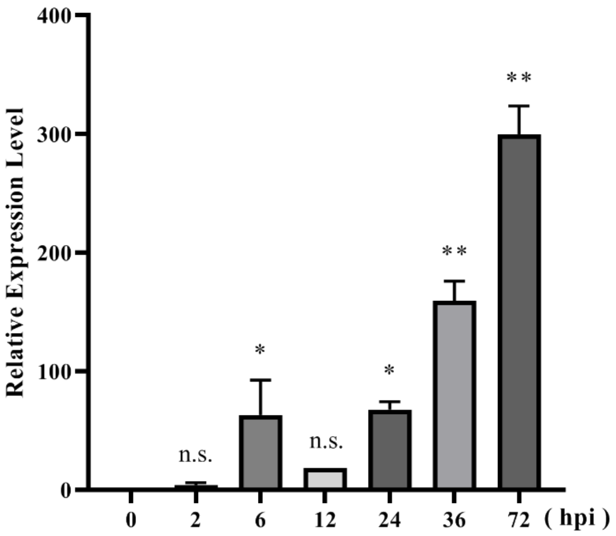

Figure S4. Expression pattern of target gene (Tae-WIP) in wheat plants after inoculation of *Bgt*. Relative expression levels are representative of the mean values of three biological replicates.Error bars represent one standard deviation (SD). The * and ** represent significant differences atlevels of $P<0.05$ and $P<0.01$ between the control and treatment groups using Tukey's multiplecomparisons test.
